# Vitamin D3: A Role in Dopamine Circuit Regulation, Diet-Induced Obesity, and Drug Consumption^1^,^2^,^3^

**DOI:** 10.1523/ENEURO.0122-15.2016

**Published:** 2016-05-19

**Authors:** Joseph R. Trinko, Benjamin B. Land, Wojciech B. Solecki, Robert J. Wickham, Luis A. Tellez, Jaime Maldonado-Aviles, Ivan E. de Araujo, Nii A. Addy, Ralph J. DiLeone

**Affiliations:** 1 Department of Psychiatry, Yale University School of Medicine, New Haven, Connecticut 06519; 2 The John B. Pierce Laboratory, New Haven, Connecticut 06519

**Keywords:** dopamine, drug addiction, feeding, obesity, vitamin D

## Abstract

The influence of micronutrients on dopamine systems is not well defined. Using mice, we show a potential role for reduced dietary vitamin D3 (cholecalciferol) in promoting diet-induced obesity (DIO), food intake, and drug consumption while on a high fat diet. To complement these deficiency studies, treatments with exogenous fully active vitamin D3 (calcitriol, 10 µg/kg, i.p.) were performed. Nondeficient mice that were made leptin resistant with a high fat diet displayed reduced food intake and body weight after an acute treatment with exogenous calcitriol. Dopamine neurons in the midbrain and their target neurons in the striatum were found to express vitamin D3 receptor protein. Acute calcitriol treatment led to transcriptional changes of dopamine-related genes in these regions in naive mice, enhanced amphetamine-induced dopamine release in both naive mice and rats, and increased locomotor activity after acute amphetamine treatment (2.5 mg/kg, i.p.). Alternatively, mice that were chronically fed either the reduced D3 high fat or chow diets displayed less activity after acute amphetamine treatment compared with their respective controls. Finally, high fat deficient mice that were trained to orally consume liquid amphetamine (90 mg/L) displayed increased consumption, while nondeficient mice treated with calcitriol showed reduced consumption. Our findings suggest that reduced dietary D3 may be a contributing environmental factor enhancing DIO as well as drug intake while eating a high fat diet. Moreover, these data demonstrate that dopamine circuits are modulated by D3 signaling, and may serve as direct or indirect targets for exogenous calcitriol.

## Significance Statement

Obesity rates have risen in this country, and low levels of circulating vitamin D3 correlate with high adiposity. Recent evidence suggests effects of vitamin D3 on dopamine circuits. While altered dopamine signaling has been implicated in the progression of obesity and the consumption of drugs of abuse, a role for vitamin D3 in these disease states has not been experimentally explored. Using reduced dietary D3, as well as treatment with the fully active form of D3, we demonstrate the effects on food and drug intake, as well as robust effects on dopamine circuits of the brain. These data suggest dopamine circuits to be a novel therapeutic target for D3 treatment with implications for obesity, drug addiction, and other dopamine-dependent behaviors.

## Introduction

Over the last 2 decades, obesity rates have increased dramatically in much of the developed world. While animal studies using single-gene mutations have contributed to our understanding of signaling pathways and neuronal circuits involved in feeding behavior and homeostasis, single-gene variants account for a small percentage of the obese populace, as weight is also influenced by many extrinsic factors (Speakman et al., 2008). Intake of high-calorie diets increases adiposity in animals, establishing an effective model for understanding the impact of energy-dense food, but research examining the causative roles of micronutrient (noncaloric) deficiencies is incomplete. Moreover, these high-calorie, nutritionally complete rodent diets do not accurately model typical Western dietary choices, which contain little to no amounts of dietary vitamin D3 (cholecalciferol; Liebman et al., 2003; Pereira et al., 2005; Jeffery et al., 2006; Keskitalo et al., 2008; García et al., 2009; Harnack et al., 2011; U.S. Department of Agriculture, 2015). Despite fortification of dairy products with vitamin D3, the typical intake for adult men and women still falls below the Institute of Medicine's recommended daily allowance of 600 IU/d Calvo et al., 2004; Cashman and Kiely, 2016).

The increased prevalence of vitamin D3 deficiency in the United States is historically concurrent with obesity rates (Mokdad et al., 2003; Ginde et al., 2009). Regardless of the source of cholecalciferol (sunlight, food, or supplementation), it must undergo the following two hydroxylation events: the first to generate the intermediate metabolite 25(OH)D3 (calcidiol); and the second to yield 1α,25(OH)_2_D3 (calcitriol), the fully active form. Circulatory levels of the intermediate 25(OH)D3 are used to determine adequacy (>20 ng/ml), and typically an inverse relationship between body mass index (BMI) and levels of calcidiol is found in obese individuals (McGill et al., 2008; Young et al., 2009). While some studies have identified polymorphisms in vitamin D3 signaling associated with obesity or fat mass (Gu et al., 2009; Vasilopoulos et al., 2013), the contribution of reduced dietary D3 on the development of obesity and overconsumption has not been directly tested in diet-induced obesity (DIO) models (Foss, 2009; García et al., 2009; Ginde et al., 2009).

The inability to regulate consumption despite adverse health consequences shares similarities with drug addiction and involves similar neural pathways (Trinko et al., 2007; Dileone et al., 2012; Volkow et al., 2013). Animal studies have demonstrated that dopamine pathways respond to the consumption of palatable foods and peripherally derived metabolic signals, and that manipulation of this circuit alters intake (Fulton et al., 2006; Hommel et al., 2006; Johnson and Kenny, 2010; Vucetic and Reyes, 2010). Moreover, amphetamine-based diet aids, as well as drugs of abuse, also target these same circuits (Ersner, 1940; Volkow et al., 2013). Vitamin D3 receptor (VDR), which functions as a transcription factor upon activation by calcitriol, is well studied in the context of bone metabolism and calcium absorption/homeostasis (Nicolaysen, 1937; Carlsson, 1952; DeLuca, 1974; McDonnell et al., 1989), but has only recently been studied for additional roles in the nervous system. The receptor is expressed in developing dopamine neurons (Cui et al., 2013), and rodent studies suggest that repeated calcitriol treatments can protect dopamine neurons against toxic doses of amphetamine, as well as enhance evoked dopamine in the striatum (Cass et al., 2006, 2012). Additional studies demonstrate that calcitriol can augment dopamine after lesioning (Cass et al., 2014). Here we provide evidence for dietary and exogenous vitamin D3 influencing dopamine circuits, responses to drugs of abuse, intake of food, and the development of obesity.

## Materials and Methods

### Animals and diet design

Animal experiments were performed in accordance with the Yale University School of Medicine Institutional Animal Care and Use Committee animal care guidelines. Dietary deficiency, microdialysis, gene expression, and amphetamine behavioral experiments were performed in male C57BL/6J mice between the ages of 8 and 10 weeks, purchased from The Jackson Laboratory. The length of time on their diets is specified within the text for specific experiments. All high fat (HF) deficiency studies used the HF diet (formulated as TestDiet 5TLN, Purina Mills) and HF-D (5TLN with reduced vitamin D3 targeting to ∼11% of normal levels; Table 1). The HF and HF-D diets were purchased from either Purina Mills LabDiet) or Harlan Teklad. All chow deficiency studies used standard rodent chow (Ch) (formulated as Prolab RMH3000, Purina Mills LabDiet) and Ch-D (Prolab RMH3000 with reduced vitamin D3 targeted to ∼7% of normal levels; Table 1). The Ch and Ch-D diets were purchased from Purina Mills LabDiet. Levels of dietary D3 used in deficient diets are high enough to prevent physiologically relevant changes in levels of serum calcium, serum phosphate, or parathyroid hormone (Anderson et al., 2007; Harnack et al., 2011; Mallya et al., 2016). Animals (for mice and rats, see Table 2) used in all treatment studies were maintained on either standard rodent Ch (RMH 3000, Purina Mills LabDiet), the HF diet, or the HF-D diet (Table 1). Immunofluorescent studies used male D1R-Cre C57BL/6J strain EY262Gsat, and male D2R-Cre C57BL/6J strain ER44Gsat transgenic mice. Mice were either group or single-housed, as specified in the text, and maintained on a 12 h light/dark cycle throughout all experiments. For fast-scan cyclic voltammetry experiments, young adult male Sprague Dawley rats (275-400 g) were acquired from Charles River Laboratories, placed on *ad libitum* food and water, and housed two to three per cage on a 12 h light/dark cycle (lights on at 7:00 A.M.).

**Table 1:** Caloric breakdown of the different diets used for these experiments, as well as dietary vitamin D3 modifications

Diet	Fat (% kcal)	Protein (% kcal)	Carbohydrate (% kcal)	Combined (kcal/g)	Dietary D3 (cholecalciferol) (IU/g)
HF	35	15	50	4.3	1.10
HF-D	35	15	50	4.3	Range: 0.092-0.12
Ch	14	26	60	3.2	2.40
Ch-D	14	26	60	3.2	0.16

The range shown for the HF-D diet represents the values observed over multiple batches.

**Table 2:** Animal usage breakdown by cohort, experiment, species, diets, and days on the respective diets

Cohort	Experiment	Species	Diets	Diet length (d)
1	Chronic deficiency, body weight, FI	Mouse, *n* = 10,10	HF, HF-D	116
(1)	Chronic deficiency, plasma	Mouse, *n* = 5, 5	HF, HF-D	165
2	Chronic deficiency, body Weight, FI	Mouse, *n* = 5, 5	Ch, Ch-D	127
3	Chronic deficiency, body weight, serum	Mouse, *n* = 5, 5	HF, HF-D	50
4	Chronic deficiency, body weight, serum	Mouse, *n* = 5, 5	Ch, Ch-D	50
5	DIO, acute calcitriol, leptin, body weight, FI	Mouse, *n* = 8, 8	HF	30
6	Acute calcitriol, CPA	Mouse, *n* = 8, 8	Std Chow	∼63
7	qPCR analysis	Mouse, *n* = 5, 5	Std Chow	∼63
8	Microdialysis	Mouse, *n* = 6, 6	Std Chow	∼63
9	FSCV	Rat, *n* = 9, 11	Std Chow	n/a
10	Chronic deficiency, amph locomotor	Mouse, *n* = 5, 5	HF, HF-D	166
(10)	Chronic deficiency, amph licking	Mouse, *n* = 5, 5	HF, HF-D	200
11	Chronic deficiency, amph locomotor	Mouse, *n* = 4, 4	Ch, Ch-D	163
12	Acute calcitriol, amph locomotor	Mouse, *n* = 8, 8	Std Chow	∼63
13	Acute calcitriol, amph licking	Mouse, *n* = 12, 12	Std Chow	∼63

amph, Amphetamine; FI, food intake; Std, standard.

### Statistics

Microsoft Excel, IBM SPSS Statistics V19, or GraphPad Prism 6.0 were used to calculate statistical tests. All *t* tests performed were two-tailed, unpaired. When applicable, ANOVAs were assessed and *post hoc* analyses were conducted. All error bars shown in the graphs represent ±SEM.

### Drugs

Calcitriol (BML-DM200, Enzo Life Sciences) was solubilized with 100% EtOH to 1 μg/μl and stored in aliquots at −80°C until used. The dilution vehicle for calcitriol injections consisted of 20% EtOH, 30% propylene glycol, and 50% H_2_O, and a working concentration of 4 ng/μl was used to minimize ethanol delivery. A single acute dose of 10 µg/kg is similar to treatments used by others and has a low toxic effect (Pakkala et al., 1995; Cekic et al., 2011; Deluca et al., 2011; Chow et al., 2013). Leptin (498-OB-01M, R&D Systems) was reconstituted with sterile alkaline 1× PBS, pH 8.2, and diluted to a 0.6 mg/ml working solution for injections. d-Amphetamine sulfate (A-5880, Sigma-Aldrich) was dissolved in sterile saline solution or water and was used immediately. The doses of amphetamine used represent the total composition (including the salt). The dopamine transporter inhibitor GBR-12909 dihydrochloride (D052-25MG, Sigma-Aldrich) was dissolved in heated saline solution.

### Deficient diet-induced obesity

Group-housed C57BL/6J male mice 8-10 weeks of age were placed on either the HF or HF-D diets *ad libitum*. Food intake and body weight were measured several times weekly. After Day 101, mice were single housed, and food intake and body weight measurements were taken daily until day 116. Mice were killed on day 164 of their diets. Single-housed C57BL/6J male mice 8-10 weeks of age were placed on either the Ch or Ch-D diets *ad libitum*. Food intake and body weight were measured several times weekly until day 101. These mice were killed on day 127 of their diets.

### Calcitriol HF diet

Single-housed naive adult male C57BL/6J mice were exposed chronically to the HF diet *ad libitum* for 26 d. On test day, the HF diet was removed from the cages, and mice were given the following two injections: (1) calcitriol (10 μg/kg body weight) or vehicle 6-7 h prior to the dark cycle; and (2) leptin (3 mg/kg body weight) or vehicle (PBS) just prior to the dark cycle, at which time the HF diet was replaced for subsequent measuring.

### Calcitriol-conditioned place aversion

Naive adult male C57BL/6J group-housed mice fed standard chow were used for conditioned place aversion (CPA). Med Associates place conditioning boxes consisting of two chambers with retractable doors were used for CPA testing. One chamber had a wire mesh floor with vertical white and black stripes on the walls, while the other chamber had a grid floor with diagonal marble and black stripes on the walls. These two chambers were separated by a gray neutral chamber. Time spent in each chamber was determined by photocell beam breaks and Med-PC IV software. Pre-exposure consisted of allowing the mice to freely explore all chambers for 20 min. Groups were determined by counterbalancing the pre-exposure bias toward a particular chamber. Conditioning on days 1 and 3 consisted of treating the mice with vehicle or 10 µg/kg calcitriol and immediately pairing the mice with a specific chamber for 4 h. Conditioning on days 2 and 4 consisted of treating with just vehicle and pairing the mice with the opposite chamber for 4 h. On day 5, mice were tested by allowing them to freely explore the boxes for 20 min. The difference in time spent in the paired chamber (postconditioning − pre-exposure) was used to evaluate CPA.

### 25-Hydroxy vitamin D3 EIA

Plasma or serum from mice was collected and stored at −80°C. EIA kit (AC-57F1 ImmunoDiagnostic Systems) was used per manufacturer instructions, and data were analyzed and graphed using Excel and Prism.

### Serum calcium analysis

Serum from trunk blood was collected and stored at −80°C. QuantiChrom Calcium Assay Kit (DICA-500, BioAssay Systems) was used per manufacturer instructions, and data were analyzed and graphed using Excel and Prism.

### Immunofluorescence

D1R-Cre C57BL/6J and D2R-Cre C57BL/6J transgenic mice were intracardially perfused using 4% paraformaldehyde. Following 30% sucrose cryoprotection and 40 µm sectioning, immunofluorescence was performed as previously described (Cui et al., 2013). Briefly, sections were pretreated in 2% Triton X-100/1× PBS for 30 min prior to blocking buffer (3% donkey serum, 0.3% Triton X-100, 1× PBS for at least 60 min). The following primary antibodies were diluted accordingly in blocking buffer, and sections were incubated overnight at room temperature with gentle shaking: tyrosine hydroxylase (TH; 1:10,000; MAB318, EMD Millipore), VDR (1:100; SC-1009 N-20, Santa Cruz Biotechnology), and Cre (1:500; MAB3120, EMD Millipore). The VDR antibody chosen was previously shown to produce nuclear staining and to generate a single band by Western blot (Cui et al., 2013). Secondary antibodies included donkey anti-rabbit (1:500; Alexa Fluor 555; Life Technologies) and donkey anti-mouse (1:500; Alexa Fluor 488).

### Calcitriol quantitative PCR

Mice were injected with either vehicle or calcitriol (10 μg/kg body weight, i.p.) and then killed by rapid decapitation 6-7 h after injection. Recovered brains were chilled for 1 min in artificial cerebral spinal fluid (aCSF) as follows (in mM): 124 NaCl, 4 KCl, 26 NaHCO_3_, 10 Glucose, 1.5 CaCl, 1.5 MgSO_4_, and 1.25 KH_2_PO_4_, pH 7.4, on ice. Coronal sections were made using razorblades and a 1 mm brain block. Using a light microscope, sections submerged in chilled aCSF were identified based on a mouse brain atlas (Paxinos and Franklin, 2004), and dissections were performed using either a 15 gauge Hamilton syringe or stab dissector while submerged in aCSF. The following coordinates relative to bregma were used: accumbens, +1.54 mm; and midbrain, −3.08 mm. Dissections were immediately recovered, frozen on dry ice, and stored at −80C for future processing. Total RNA was extracted using TRIzol Reagent (Ambion/Life Technologies) per manufacturer instructions and was quantified by Nanodrop. Following cDNA generation, samples were assessed for changes in gene expression using either Taqman (Applied Biosystems/Life Technologies) or SybrGreen (Applied Biosystems/Life Technologies) based reporters. Primer pairs using SybrGreen were designed to cross intron–exon boundaries, and underwent efficiency curves for validation prior to experimental use. Analysis was based on the 2^-ΔΔCt^ method (Livak and Schmittgen, 2001) . TATA Box Binding Protein (*Tbp*) primer pair was used as the loading control for all quantitative PCR (qPCR) data. Taqman gene assays included *Tbp* (Mm0044697[lowem]m1), dopamine receptor type 2 (*Drd2*; Mm00438545[lowem]m1), and *Th* (Mm00447557[lowem]m1). See Table 3 for primers using SybrGreen.

**Table 3:** Primers using SybrGreen

Gene	Primer	
*Drd1*	*Forward*	*5'-GAGCGTGGTCTCCCAGAT-3'*
*Drd1*	*Reverse*	*5'-TCACTTTTCGGGGATGCT-3'*
*Slc6a3*	*Forward*	*5'-CTGGTGCTGGTCATTGTTCT-'*
*Slc6a3*	*Reverse*	*5'-AGCAGGGCTGTGAGGACTAC-3'*
*Oprm1*	*Forward*	*5'-CCATCATGGCCCTCTATTCT-3'*
*Oprm1*	*Reverse*	*5'-TGTTGGTGGCAGTCTTCATT-3'*
*Tbp*	*Forward*	*5'-AAAGGGAGAATCATGGACCAGAACAA-3'*
*Tbp*	*Reverse*	*5'-TGGACTAAAGATGGGAATTCCAGGAG-3'*

### Locomotor activity assay

All mice were habituated to the locomotor chambers (Med Associates) for 90 min prior to d-amphetamine sulfate treatment (2.5 mg/kg). For the HF and HF-D deficiency study, testing occurred on day 166 of their diets, and 1 h into the habituation phase they received an intraperitoneal injection of saline, and 30 min later received amphetamine. For the Ch and Ch-D deficiency study, testing occurred on day 163 of their diets, and 1 h into the habituation phase they received an intraperitoneal injection of saline, and 30 min later received amphetamine. For the calcitriol treatment study, naive mice fed the Ch diet over the long term were pretreated with either vehicle or calcitriol (10 μg/kg) 6-7 h prior to placement in locomotor boxes, and no saline was delivered during their habituation. After habituation, amphetamine (2.5 mg/kg, i.p.) was administered to all mice, and locomotor activity was recorded in 10 min bins.

### Oral amphetamine

Lickometer boxes (Med Associates) containing two separate but identical spigots (labeled A and B) located on the same wall were used for training and testing. Licks on both spigots were electronically monitored in real time using Med-PC IV software, and training sessions occurred every other day. The night prior to all training sessions, mice were water deprived to induce drinking. All training sessions were conducted for 1 h during the light cycle with both spigots in place, but with only one delivering a liquid. The first training session involved only water delivered by spigot B, while the subsequent two training sessions involved only d-amphetamine sulfate dissolved in tap water (90 mg/L, made fresh daily) in spigot A. This dose is similar to those used by others for oral consumption (Kamens et al., 2005; Eastwood et al., 2014). After each training session, the mice were placed back in their home cage with access to water and food. After three training sessions, the mice were tested over 18 h starting at the dark cycle with both water and amphetamine spigots loaded, as well as appropriate food pellets. For the HF and HF-D deficiency study, the same cohort of mice used for the locomotor amphetamine study was used on day 200 of their diets. For the calcitriol study, 6-7 h prior to initiation of the test session, mice that had been maintained on the Ch diet were injected with either vehicle or calcitriol (10 μg/kg).

### Microdialysis

Mice were anesthetized with an injection of a ketamine/xylazine (100/15 mg/kg, i.p.) mix, and a small incision was made into the lower lateral abdominal area into which the tip of MicroRenathane tubing (Braintree Scientific) was inserted. A purse string was tightened around the tubing, which was then tunneled subcutaneously to the dorsum via a small hole made into the abdominal muscle. A small incision between the shoulder plates was then made on the dorsum to allow for catheter exteriorization. Incisions were sutured and thoroughly disinfected, and the exterior end of the catheter was plugged. Immediately after the above procedure, the mouse was placed on a stereotaxic apparatus (David Kopf Instruments) under constant flow of ∼1% isoflurane anesthesia (1.5 L/min), and a small circular hole was drilled [position from brain surface: anteroposterior (AP), 1.3 mm; mediolateral (ML), ±1.3 mm] for implantation of a guide cannula [dorsoventral (DV), −0.5 mm from brain surface] for posterior insertion of a microdialysis probe into the dorsal striatal region (final probe tip positions: DV, −2.5 mm from brain surface). Microdialysis measurements were performed as in our previous studies (Ren et al., 2010; Ferreira et al., 2012). Specifically, during the experimental sessions, microdialysate samples from the freely moving mice were collected, separated, and quantified by HPLC coupled to electrochemical detection methods. Briefly, after recovery from surgery, a microdialysis probe (cutoff, 6kDa; 2-mm-long; CMA-7, CMA Microdialysis) was inserted into the striatum through the guide cannula (the corresponding CMA-7 model). After insertion, probes were connected to a syringe pump and perfused at 1.3 µl/min with aCSF (Harvard Apparatus). After a 60 min washout period, dialysate samples were collected every 10 min and immediately manually injected into an HTEC-500 HPLC unit (Eicom). Analytes were then separated via an affinity column (PP-ODS, Eicom), and compounds were subjected to redox reactions within an electrochemical detection unit (amperometric DC mode; applied potential range, 0 to ∼2000 mV; 1 mV steps). Resulting chromatograms were analyzed using EPC-300 software (Eicom), and actual sample concentrations were computed based on peak areas obtained from a 0.5 pg/µl dopamine standards (Sigma-Aldrich) and expressed as percentage changes with respect to the mean dopamine concentration associated with baseline (i.e., previous to vehicle or amphetamine infusions) samples. Locations of microdialysis probes were confirmed histologically. Calcitriol (10 μg/kg) or vehicle was delivered via catheter ∼6-7 h prior to amphetamine injection (2.5 mg/kg). After the establishment of a stable baseline over 30 min, the mice were treated with amphetamine.

### Fast-scan cyclic voltammetry

Rats were pretreated with calcitriol (10 μg/kg) or vehicle 6 h prior to surgery. Rats were anesthetized with urethane (1.5 mg/kg) and placed in a stereotaxic frame (David Kopf Instruments). A bipolar, stainless steel stimulating electrode (Plastics One) was placed into the ventral tegmental area (VTA)/substantia nigra [SN; AP, −5.2 mm; ML, 0.5-1.5 mm; DV, 7.4-8.1 mm below dura (Paxinos and Watson, 2007)], a Ag/AgCl reference electrode was placed into the contralateral cortex, and a carbon ﬁber microelectrode was implanted in the dorsal striatum (AP, +1.2 mm; ML, −1.4 mm; DV, 4.0–5.0 mm). During fast-scan cyclic voltammetry (FSCV) recording, body temperature was maintained at 37°C by a heating pad (Harvard Apparatus).

T-650 carbon ﬁbers (Thornel, Amoco Corp.) were aspirated into a glass capillary and pulled with an electrode puller (Narishige International). The carbon fiber protruding from the capillary was then cut under a light microscope to a length of 100–200 µm. A triangular waveform (−0.4 to +1.3 V and back to −0.4 V vs an Ag/AgCl reference electrode) was applied at 400 V/s and repeated at 100 ms intervals. The triangular waveform was low-pass ﬁltered at 2 kHz. Data were digitized and processed using NI-6711 and NI-6251 PCI cards (National Instruments), and were analyzed using Demon Voltammetry and Analysis Software (Wake Forest Baptist Medical Center) or Tar Heel CV Software (The University of North Carolina at Chapel Hill, Chapel Hill, NC). Background-subtracted cyclic voltammograms were obtained by digitally subtracting stable background currents to resolve cyclic voltammograms associated with the electrical stimulation event. To evoke phasic dopamine release in the dorsal striatum, electrical stimulation (300 µA, 60 pulses, 2 ms each phase at 60 Hz) was applied to the VTA. Pulses delivered to the stimulating electrode were computer generated with a 6711 PCI card (National Instruments) and were optically isolated from the electrochemical system using a biphasic stimulus isolator (model NL 800A NeuroLog System, Digitimer). Each stimulation train was applied every 5 min to allow time for dopamine-releasable stores to return to their original levels. Baseline evoked phasic dopamine release consisted of at least three stimulations over a period of 15 min prior to injecting the drug. Evoked phasic dopamine release recorded after intraperitoneal injection of either amphetamine (2.5 mg/kg) or GBR-12909 dihydrochloride (15 mg/kg) was normalized to baseline for each animal.

## Results

### High fat diet with reduced D3 enhances body weight gain and food intake

**Figure 1.**
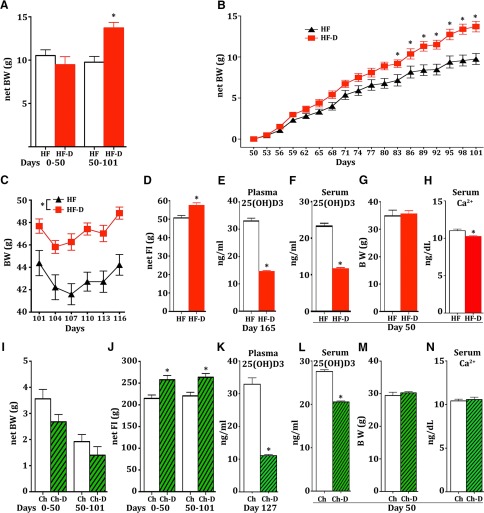
Vitamin D3 levels modulate food intake (FI) and body weight (BW). ***A***, HF and HF-D mice exhibited no difference in net body weight between groups over the first 50 d of diet exposure, but a significant increase for the HF-D mice was observed for days 50-100 (*n* = 10, 10). ***B***, Binned change in body weight for HF and HF-D groups from day 50 to day 101 (*n* = 10, 10). ***C***, HF and HF-D mice maintained significant differences in body weight throughout the transition from group-housed to single-housed environment from day 101 to day 116 (*n* = 5, 5). ***D***, Cumulative food intake for HF and HF-D groups from day 101 to day 116 (*n* = 5, 5). ***E***, Plasma levels of 25(OH)D3 from the HF and HF-D mice on day 165 of their diets (*n* = 5, 5) used in ***C*** and ***D***. ***F***, ***G***, A separate cohort of HF and HF-D mice (*n* = 5, 5) was killed on day 50 of their diets (***F***), and serum analysis of 25(OH)D3 levels revealed a significant reduction, despite no differences in body weight (***G***). ***H***, These mice exhibited a small yet significant reduction in serum calcium levels (*n* = 5,5). ***I***, Single-housed mice chronically exposed to Ch or Ch-D diets had no significant difference in body weight between groups over days 0-50 or 50-101 (*n* = 5,5). ***J***, These mice did, however, exhibit increased food intake over both time periods (*n* = 5, 5). ***K***, At the end of this experiment on day 127, the Ch-D mice were found to have reduced plasma levels of 25(OH)D3. ***L***, A separate cohort of group-housed Ch and Ch-D mice (*n* = 5, 5) was killed on day 50, and were found to have significantly reduced serum 25(OH)D3 levels. ***M***, These mice displayed no differences in body weight (*n* = 5, 5). ***N***, Additionally, no differences in serum calcium levels were detected (*n* = 5, 5).

To evaluate the effects of reduced dietary D3, an HF diet was modified (i.e., the HF-D diet) to have only ∼11% of the D3 levels present in the HF diet (Table 1). Group-housed mice were fed either the HF-D or HF diet *ad libitum*. Over the first 50 d, no difference in body weight between groups was observed; however, over the following 50 d, the HF-D group gained significantly more weight (Fig. 1*A*, *p = 0.0005, *t* test, *t* = 4.239). Binned data during this period revealed a persistent increase in weight that was significant across 21 d [Fig. 1*B*, two-way ANOVA, treatment * time (*F*_(17,306)_= 8.397, *p < 0.0001), Bonferroni’s *post hoc* test]. Mice were then single housed to measure food intake. Over the next 16 d, the groups maintained a significant difference in body weight (main effect of diet: *F*_(1,8)_ = 15.87, *p = 0.004, Bonferroni’s *post hoc* test), and a significant increase in food intake for the HF-D group was observed (Fig. 1*C*,*D*, *p = 0.0106, *t* test, *t* = 3.316). To verify the effect of dietary manipulation, plasma 25(OH)D3 was assessed at the end of the experiment, and the expected reduction was found in the HF-D group (Fig. 1*E*, *p < 0.0001, *t* test, *t* = 16.10). An additional cohort of mice was generated and killed on day 50. Serum 25(OH)D3 level was found to be significantly lower in these HF-D mice compared with HF mice despite no change in body weight at this time point, suggesting that lower levels of D3 precede increases in body weight (Fig. 1*F*,*G*, *p < 0.0001, *t* test, *t* = 12.64). Serum calcium levels were found to be significantly reduced (Fig. 1*H*, *p = 0.0131, *t* test, *t* = 3.176).

### Standard chow diet with reduced D3 enhances food intake

To evaluate the effects of reduced dietary D3 on body weight and food intake with standard chow, a Ch diet was modified (i.e., Ch-D diet) to have ∼7% of the Ch diet (Table 1). Single-housed mice were fed either Ch-D or Ch diets *ad libitum*. No differences in body weight were observed from days 0-50 or 50-101 (Fig. 1*K*). A significant increase in food intake was observed, however, for these time periods (Fig. 1*K*: days 0-50: *p = 0.0091, *t* test, *t* = 3.416; days 50-101: *p = 0.0069, *t* test, *t* = 3.606). To verify the effect of dietary manipulation, plasma 25(OH)D3 level was assessed at the end of the experiment, and the expected reduction was found in the Ch-D group (Fig. 1*K*, *p < 0.0001, *t* test, *t* = 11.04). An additional cohort of Ch- and Ch-D-fed mice was generated and killed on day 50. The serum 25(OH)D3 level was found to be significantly lower in these Ch-D-fed mice compared with HF-fed mice despite no change in body weight at this time point, suggesting that lower levels of D3 precede increases in food intake (Fig. 1*L*,*M*, *p < 0.0001, *t* test, *t* = 14.85). Serum calcium was found to be unchanged between groups (Fig. 1*N*).

### Calcitriol reduces body weight and food intake in obese mice

**Figure 2.**
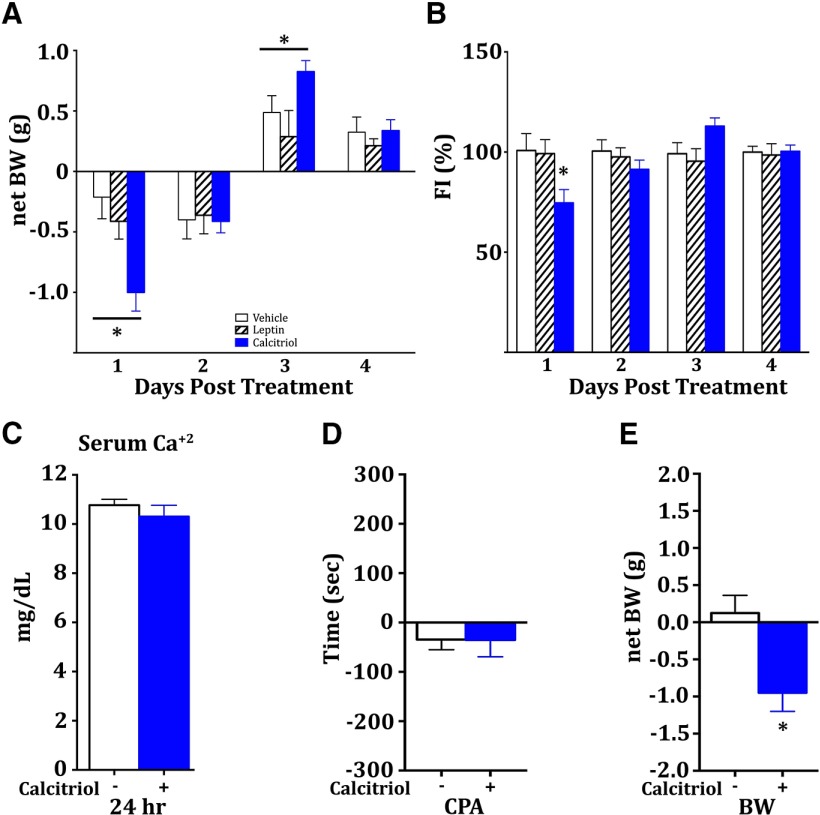
***A***, After 26 d of chronic HF exposure, naive mice were treated with vehicle, calcitriol, or leptin, and displayed reduced net body weight in response only to calcitriol for the first day after single acute treatment (*n* = 8, 8, 8). ***B***, Calcitriol treatment also reduced food intake for the first day after treatment in these mice (*n* = 8, 8, 8). ***C***, An additional long-term HF exposure cohort was generated to assess the effects of acute calcitriol treatment on serum calcium levels. After 32 d of the HF diet, mice were treated in the short term and killed 24 h later. No changes in serum calcium levels were observed (*n* = 8, 8). ***D***, CPA was performed on a separate cohort of naive mice to assess potential aversive effects of calcitriol that could contribute to altered feeding behavior. ***E***, After 4 d of alternating conditioning treatments to specific chambers, there was no difference between groups in the change in the amount of time spent in the paired chamber, yet a significant reduction in net body weight was observed (*n* = 8, 8). All error bars indicate the SEM.

Diet-induced obesity in both humans and animals results in increased adiposity, as well as the gradual failure of the anorexigenic hormone leptin to regulate intake and metabolic homeostasis, a phenomenon known as leptin resistance (Van Heek et al., 1997). To complement the dietary deficiency experiments, we assessed the potential effects of exogenous calcitriol on reducing HF food intake after establishing DIO in mice. After being placed on the HF diet for 26 d, mice were treated with vehicle or a single acute dose of calcitriol (10 µg/kg) 6-7 h prior to the dark cycle, or a single dose of leptin (3 mg/kg) just prior to the dark cycle. The 26 d of eating the HF diet generated leptin-resistant mice, demonstrated by the inability of leptin to reduce either body weight or food intake (Fig. 2*A*,*B*). In contrast, acute administration of calcitriol caused a net reduction in body weight, which rebounded by day 3 post-treatment [Fig. 2*A*, two-way ANOVA, treatment * time (*F*_(6,63)_ = 3.148, *p* < 0.01), Tukey’s *post hoc* test], and a reduction in food intake [Fig. 2*B*, two-way ANOVA, treatment * time (*F*_(6, 63)_ = 3.200, *p < 0.01) Tukey’s *post hoc* test] over the first 24 h. These results demonstrate that exogenous calcitriol may have therapeutic benefits in affecting body weight and food intake under conditions of obesity and leptin resistance. To assess a potential calcemic contribution to these effects, an additional group of mice placed on the long-term HF diet were treated with exogenous calcitriol (10 µg/kg) or vehicle and were killed after 24 h for serum collection. Calcium was found to be unchanged at this time point after treatment (Fig. 2*C*). Reduced food intake and body weight following pharmacological treatments can be due to indirect aversive effects by the treatments; however, conditioned place aversion tests can be performed to evaluate this effect. Mice were conditioned with vehicle or calcitriol (10 µg/kg) to a paired chamber over 4 d (4 h each day, every other day), and then tested for changes in exploratory behavior. No differences between groups were observed for time spent in the paired chamber after conditioning, suggesting that there is no aversive effect contributing to reduction of food intake (Fig. 2*D*). A significant difference in body weight was observed in the CPA mice as a result of treatment, demonstrating that calcitriol was effective (Fig. 2*E*, *p = 0.0075, *t* test, *t* = 3.123).

### Dopamine circuits express VDR and respond to calcitriol

**Figure 3.**
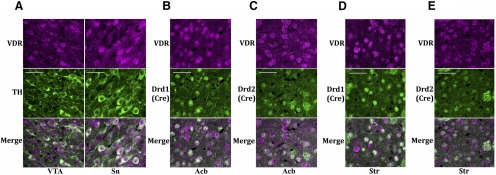
VDR is expressed in dopamine-producing, as well as in dopamine-receiving, neurons. ***A***, Colocalization of TH (green) with VDR (purple) in subregions of the midbrain including the VTA and SN. White scale bars indicate a distance of 40 µm. ***B–E***, Colocalization of Cre recombinase (green) with VDR (purple) using the D1R-Cre and D2R-Cre transgenic mouse lines in the nucleus accumbens (Acb; ***B***, ***C***), as well as in the dorsal striatum (Str; ***D***, ***E***).

**Figure 4.**
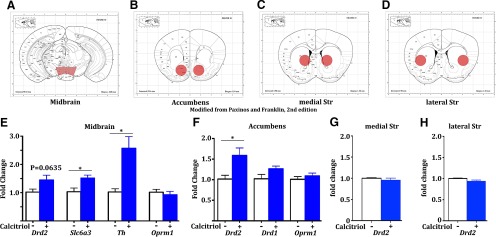
Calcitriol treatment modulates gene expression differentially throughout dopamine circuitry. ***A–D***, Mouse brain atlas diagrams (Paxinos and Franklin, 2004) showing typical dissections for qPCR analysis (red areas). ***E–H***, Naive mice treated acutely with vehicle (−) or calcitriol (+; *n* = 5, 5) 6-7 h prior to being killed revealed differential effects of calcitriol in the midbrain, accumbens, and medial and lateral striatum (Str), as assessed by qPCR. All error bars indicate the SEM.

To evaluate the expression of VDR in dopamine circuitry, we performed immunofluorescence in mice. We observed high colocalization of VDR with TH, a marker for neurons in the ventral tegmental area and substantia nigra that produce dopamine (Fig. 3*A*), as well as expression in non-TH neurons, which is consistent with findings of a recent study (Cui et al., 2013). Using transgenic mice to identify dopamine type 1 or type 2 receptors, we also found VDR to be highly colocalized with both cell types in the nucleus accumbens (Fig. 3*B*,*C*, “Acb”), as well as the dorsal striatum (Fig. 3*D*,*E*). Virtually all (>95%) Cre-positive neurons expressed VDR. Since VDR functions as a transcription factor (McDonnell et al., 1989), we assessed gene expression changes in these regions. Naive mice were injected with calcitriol 6-7 h prior to being killed, dopamine-related brain regions were dissected (Fig. 4*A–D*), and genes of interest were analyzed by qPCR (*n* = 5, 5 for all treatments). In the midbrain, we observed significant upregulation of *Th* (*p = 0.0067, *t* test, *t* = 3.626) and dopamine transporter (*Slc6a3*: *p = 0.0174, *t* test, *t* = 2.986), as well as a trend increase in *Drd2* (*p* = 0.0635) and no effect on μ-opioid receptor (*Oprm1*; Fig. 4*E*). In the nucleus accumbens, we found significant upregulation of *Drd2* (*p = 0.0224, *t* test, *t* = 2.824), but no change in *Drd1* or *Oprm1* (Fig. 4*F*), while *Drd2* was not changed in either the medial or lateral dorsal striatum (Fig. 4*G*,*H*).

### Calcitriol enhances dopamine release

**Figure 5.**
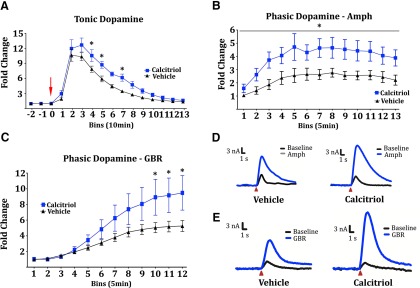
Calcitriol pretreatment enhanced dopamine release in mice and rats in response to amphetamine or dopamine transporter inhibition. All animals received acute pretreatment with calcitriol or vehicle 6-7 h prior to subsequent treatments. ***A***, Microdialysis measuring tonic dopamine release in naive mice (*n* = 6, 6) revealed the effects of calcitriol on amphetamine-induced dopamine release (red arrow, amphetamine treatment). ***B***, ***C***, FSCV experiments in rats revealed the effects of calcitriol on evoked dopamine release upon treatment with amphetamine (calcitriol, *n* = 5; vehicle, *n* = 4) or dopamine transporter inhibition (GBR-12909 dihydrochloride; calcitriol, *n* = 4; vehicle *n* = 7). ***D***, ***E***, Representative traces for FSCV experiments. All error bars indicate the SEM.

To measure neurochemical effects of acute D3 signaling on amphetamine-induced dopamine release, microdialysis probes were surgically implanted in the striatum of naive mice. Calcitriol or vehicle was administered 6-7 h prior to amphetamine treatment, and dialysate was collected and analyzed for dopamine in 10 min bins. Amphetamine (2.5 mg/kg) induced a robust dopamine increase in all animals, and calcitriol treatment significantly enhanced this effect [Fig. 5*A*, two-way ANOVA, treatment * time (*F*_(15,150)_ = 1.888, *p* = 0.0285), Bonferroni’s *post hoc* test]. To complement this, FSCV was used to measure evoked, phasic dopamine release in the striatum. Rats were pretreated with calcitriol or vehicle 6-7 h prior to amphetamine treatment. After amphetamine treatment, midbrain neurons were stimulated using a 60 Hz, 60 pulse train applied every 5 min. Similar to what was seen in the mouse microdialysis experiment, rats injected with calcitriol showed a potentiation of evoked dopamine response to amphetamine [Fig. 5*B*, *main effect of treatment (*F*_(1,7)_ = 5.746, *p* = 0.0477)]. A separate group of rats received the dopamine transporter (DAT) antagonist GBR-12909 dihydrochloride and showed an increase in evoked dopamine release, revealing that the dopaminergic effects of DAT inhibition are also altered by vitamin D3 [Fig. 5*C*, two-way ANOVA, treatment * time (*F*_(11,99)_ = 4.510, *p < 0.0001), Bonferroni’s *post hoc* test]. Representative traces for the voltammetry experiments are provided (Fig. 5*D*,*E*).

### Vitamin D3 levels alter amphetamine-induced activity

**Figure 6.**
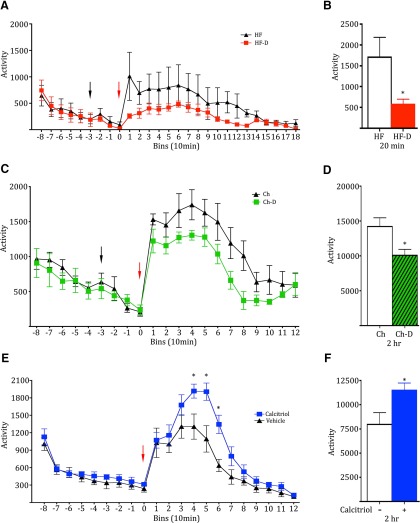
Vitamin D3 levels alter amphetamine-related behaviors in mice. ***A***, Binned locomotor activity of HF and HF-D mice during habituation, saline injection (black arrow), and acute amphetamine treatment (red arrow). ***B***, HF and HF-D cumulative activity for 20 min after amphetamine administration (*n* = 5, 5). ***C***, ***D***, Binned locomotor activity of Ch and Ch-D mice during habituation, saline injection (black arrow), and acute amphetamine treatment (red arrow; ***C***), and Ch and Ch-D cumulative activity for 2 h after amphetamine (*n* = 4, 4; ***D***). ***E***, Binned locomotor activity in naïve mice pretreated with vehicle or calcitriol (*n* = 8, 8) 6-7 h prior to acute amphetamine treatment (red arrow). ***F***, Cumulative activity of the pretreated mice over 2 h after amphetamine administration.

These molecular and neurochemical effects on dopamine systems would be predicted to alter behavioral responses to drugs of abuse, such as amphetamine. HF-D and HF mice were injected with amphetamine on day 166 of their diets and were assessed for changes in locomotor activity. No difference was observed during the habituation phase; however, the HF-D-deficient mice showed attenuated activity after amphetamine treatment (Fig. 6*A*,*B*, red arrow, *p = 0.05, *t* test, *t* = 2.293). This experiment was also conducted using Ch and Ch-D mice to avoid the effects of body weight changes. Ch and Ch-D mice were injected with amphetamine on day 163 of their diets and assessed for changes in locomotor activity. Again, no difference was observed during the habituation phase, yet the Ch-D-deficient mice displayed attenuated activity after amphetamine treatment (Fig. 6*C*,*D*, red arrow, *p = 0.0345, *t* test, *t* = 2.722). To complement these deficiency studies, naive mice were pretreated with either calcitriol or vehicle 6-7 h prior to amphetamine treatment. Again, no difference in activity was observed during the habituation phase; however, calcitriol treatment enhanced the amphetamine-induced locomotor effects [Fig. 6*C*, red arrow; two-way ANOVA, treatment * time (*F*_(11,154)_ = 2.831, *p = 0.0021), Bonferroni’s *post hoc* test], and cumulative activity over 2 h after amphetamine injection (Fig. 6*D*, *p = 0.029, *t* test, *t* = 2.442). This behavioral result is consistent with the neurochemical analysis demonstrating increased dopamine signaling as a result of calcitriol administration (Fig. 5).

### Vitamin D3 levels alter amphetamine consumption

**Figure 7.**
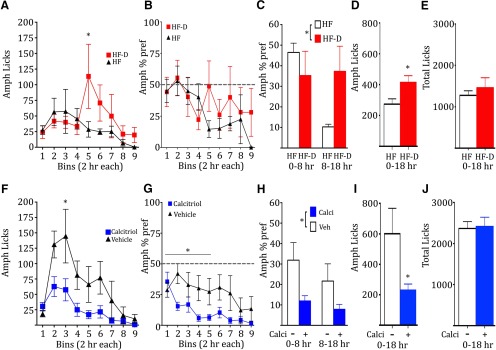
***A***, Oral amphetamine licks for HF and HF-D groups shown in 2 h bins across the 18 h test period (*n* = 5, 5). ***B***, HF and HF-D group amphetamine preferences shown in 2 h bins across the 18 h test period. ***C***, HF and HF-D amphetamine preference scores grouped for 0-8 and 8-18 h. ***D***, ***E***, Cumulative amphetamine licks for the HF and HF-D mice over the 18 h test period, as well as the total number of licks (amphetamine plus water). ***F***, Oral amphetamine licks for naive mice after single acute calcitriol or vehicle treatment (6-7 h prior to testing) shown in 2 h bins across the 18 h test period (*n* = 12, 12). ***G***, Amphetamine preference for the calcitriol- and vehicle-treated mice shown in 2 h bins across the 18 h test period. ***H***, Calcitriol and vehicle amphetamine preference scores grouped for 0-8 and 8-18 h. ***I***, ***J***, Cumulative amphetamine licks over the 18 h test period, as well as the total number of licks (amphetamine plus water). All error bars indicate the SEM.

Finally, to explore the potential effects of deficiency and treatment in the context of drug intake, mice were trained over several daily sessions to orally consume amphetamine (90 mg/L) or water, and individual licks were recorded. On the test day, intake was monitored over 18 h of *ad libitum* consumption of amphetamine and water, and licks were binned every 2 h. The HF-D mice displayed an increased number of amphetamine licks [Fig. 7*A*, two-way ANOVA, *main effect of treatment (*F*_(1,8)_ = 6.326, *p* = 0.0361), Bonferroni’s *post hoc* test]. Analysis of the percentage preference for amphetamine [% = 100 * (amphetamine licks/(amphetamine + water licks)] reveals that both HF and HF-D mice displayed equal preference for the initial part of the test session (Fig. 7*B*). Grouped analysis of the percentage preference found that, while there was no difference in preference for the first 8 h, the HF-D mice maintained their preference for the next 10 h, while the HF control mice exhibited reduced preference [Fig. 7*C*, two-way ANOVA, treatment * time (*F*_(1,8)_ = 18.28, *p* = 0.0027)]. HF-D mice also had significantly more cumulative amphetamine licks over the 18 h test than the HF mice (Fig. 7*D*, *p = 0.035, *t* test, *t* = 2.534). No difference in the total number of licks (amphetamine plus water) was observed between groups, suggesting that this effect was not an indirect result of an overall increase in liquid consumption (Fig. 7*E*). Complementary to the deficiency study, naive mice trained in the same manner were pretreated with calcitriol or vehicle 6-7 h prior to testing. Mice treated with calcitriol displayed a reduced number of amphetamine licks [Fig. 7*F*, two-way ANOVA treatment * time (*F*_(8,176)_ = 11.57, *p = 0.0127), Bonferroni’s *post hoc* test]. No difference in percentage of the amphetamine preference was observed for the first 2 h bin; however, calcitriol enhanced the reduction in preference over the first five bins (Fig. 7*G*, two-way ANOVA, treatment * time, *F*_(4,88)_ = 2.724, *p* = 0.0344). Due to lack of activity (sleeping) by mice in both groups toward the end of the test session, the statistical analysis only included bins 1-5. Grouped analysis for 0-8 and 8-18 h revealed a significant reduction in preference in the calcitriol group (Fig. 7*H*, two-way ANOVA, *main effect of treatment, *F*_(1,44)_ = 7.243, *p* = 0.01). Calcitriol-treated mice had significantly fewer cumulative amphetamine licks over the 18 h test period (Fig. 7*I*, *p = 0.0423, *t* test, *t* = 2.155), and there was no difference in the total number of licks between groups (Fig. 7*J*).

## Discussion

The data presented here suggest a potential role for chronic dietary D3 deficiency, specifically in the context of a high fat diet, in the development of obesity and increased drug consumption. Interestingly, the effects of deficiency on body weight occur only with a high fat diet, suggesting an interaction that may be relevant to obesity. The acute effects of calcitriol treatment on reducing both body weight and drug consumption suggest an influence of vitamin D3 signaling in behaviors mediated by dopamine circuit function. The increased intake and weight gain with the long-term HF-D diet provide evidence that a reduction, as opposed to a depletion, of dietary D3 in animals ingesting a high fat diet promotes an enhanced obesity phenotype. This is in contrast to weight loss, reduced intake, and the development of rickets that occurs gradually under prolonged conditions of complete dietary vitamin D3 depletion (Dodds and Cameron, 1943; Brommage and DeLuca, 1984). Notably, reducing circulating 25(OH)D3 levels to ∼70% does not change body weight (data not shown), suggesting that our present diet (∼50% reduction in circulating levels) is just below the threshold for effects on feeding behavior and weight. The long half-life of vitamin D3 (Smith and Goodman, 1971) likely explains the delayed and gradual body weight effects of the high fat dietary deficiency. The reduced 25(OH)D3 levels found in the 50 d HF-D cohorts, despite the lack of effects on body weight, are consistent with the deficiency preceding, and contributing to, the enhanced obesity phenotype. While a small yet significant reduction in serum calcium levels was observed in the 50 d HF-D cohort, there was no difference in serum calcium levels between the Ch and Ch-D mice at day 50, despite having a more pronounced reduction in 25(OH)D3 levels. These data contrast with previous models that suggested that vitamin D3 reduction was secondary to increased adiposity (Wortsman et al., 2000; Drincic et al., 2012), and is more consistent with a potential role for deficiency as a factor contributing to obesity in humans (Foss, 2009).

Despite the acceptance of a strong inverse relationship of 25(OH)D3 with BMI, most clinical trials using cholecalciferol supplementation in obese individuals have reported negative data for changes in BMI (Aguirre Castaneda et al., 2012; Belenchia et al., 2013; Mason et al., 2014). It is notable that, to our knowledge, there have been no clinical studies evaluating the effect of exogenous calcitriol specifically on BMI in obese patients. In the present experiments, the use of calcitriol provides rapid effects by bypassing the enzymatic steps needed for cholecalciferol conversion to calcitriol, which could be compromised in states of obesity (Barchetta et al., 2012). Moreover, the ability of acute calcitriol treatment to reduce food intake and body weight after a chronic HF diet, suggests that increased VDR signaling can have anorexigenic effects under conditions where animals are resistant to leptin. The lack of effect on general activity during habituation, total liquid consumption, and CPA data argue against general malaise or aversive effects contributing to reduced food intake and body weight after treatment. The findings of recent studies demonstrating attenuated body weight gain in rodents treated over the long term with calcitriol throughout their exposure to a HF diet (Yin et al., 2012; Alkharfy et al., 2013) complement the data presented here. Our studies expand on these findings to demonstrate effectiveness after the development of DIO, as well as potential neural mechanisms mediating the effects of acute calcitriol treatment on both food intake and body weight.

Similar to effects on food intake, mice with reduced levels of dietary vitamin D3 show increased amphetamine consumption. These chronic deficiency data are consistent with proposed models of “reward deficiency,” whereby reduced dopamine signaling, in animals and humans, leads to compensatory consumption of palatable foods or drugs (Koob and Le Moal, 1997; Wang et al., 2001; Blum et al., 2012). The attenuated locomotor response to acute amphetamine administration in both the HF-D and Ch-D mice suggests a blunted dopaminergic response as a result of the deficiency. Other research using obesity-prone rats has demonstrated reduced evoked dopamine release, while chronic intake of a high fat diet also leads to reduced dopamine release in the nucleus accumbens, as well as reduced locomotor responses to acute amphetamine treatment (Geiger et al., 2008; Geiger et al., 2009). Clearly, there are many factors (e.g., genetics, diet, other experiences) that can influence the dopamine system. The deficiency data presented here suggest an additional contributing noncaloric dietary factor leading to altered dopamine responses. Moreover, the effect of deficiency on body weight was observed only with a high fat diet, suggesting a unique interaction with vitamin D deficiency that is not observed with standard chow diets. The lack of change in body weight in the Ch and Ch-D mice is consistent with previous studies using standard chow diets (Dodds and Cameron, 1943; Brommage and DeLuca, 1984; Lagishetty et al., 2010).

Behavioral data from calcitriol treatments complement the deficiency data. Acute calcitriol treatments reduced amphetamine consumption, while also enhancing neurochemical and behavioral responses to acute amphetamine administration. This “reward sufficiency” caused by a calcitriol-induced increase in dopamine response may lead to higher sensitivity to drugs or palatable foods, thus potentially reducing consumption. While the deficiency and acute treatment studies generated opposing phenotypes, it is important to understand the limits of interpretation. These deficiency studies were performed under chronic conditions, and the mechanisms by which chronic dietary vitamin D3 deficiency contributes to the observed phenotypes may be different than the mechanisms by which exogenous treatment with calcitriol leads to the opposite effects. Additionally, it should be mentioned that increased sensitivity to drugs would be predicted to enhance intake in some contexts, while reducing it in others (Kamens et al., 2005; Robinson and Berridge, 2008; Vanderschuren and Pierce, 2010).

Other rodent research using vitamin D3 manipulations has shown similar effects on dopamine (Cass et al., 2012), while development and adult deficiency studies have yielded contrasting results (Kesby et al., 2010; Groves et al., 2013). Experimental design differences, such as the degree of dietary deficiency or treatment may account for these results. It is important to note that the amphetamine licking experiments could reflect the effects of both vitamin D3 signaling on taste responses as well as on dopamine circuit responses to amphetamine. The decrease in amphetamine preference to water in the vehicle-treated nondeficient mice is consistent with the results of other studies evaluating amphetamine consumption over time (Kamens et al., 2005; Eastwood et al., 2014). Both HF and HF-D mice initially displayed an identical preference for amphetamine, suggesting that taste responses are not likely accounting for differences in intake. The rapid decrease in preference by the calcitriol-treated mice supports the concept of increased dopamine sensitivity. In the context of HF and HF-D diets, it is notable that the HF-D mice displayed a higher preference for amphetamine, later in the session, compared with HF controls, as well as greater consumption. For both studies, the differences that developed over time are more likely due to the effects of the vitamin D3 manipulations on the dopamine circuit response to amphetamine, either reducing sensitivity or enhancing it.

The calcitriol-induced upregulation of the *Th* gene, a key enzyme in dopamine synthesis, as well as *Slc6a3*, the dopamine transporter, suggests presynaptic effects on dopamine production and reuptake, while the upregulation of *Drd2* mRNA in nucleus accumbens illustrates the potential postsynaptic effects on downstream dopamine signaling. Amphetamine and GBR-12909 dihydrochloride both increase synaptic dopamine levels, partly by targeting the dopamine transporter (Sulzer et al., 1993; Tsukada et al., 2000). Microdialysis and FSCV experiments using these compounds verified that exogenous calcitriol enhanced dopamine release, suggesting that the effect of calcitriol treatment on transcriptional regulation may underlie the physiological changes as well as the behavioral effects. The generation of complementary data with microdialysis and FSCV using two different rodent species highlights the potential conservation of this effect on dopamine release.

The changes in *Th* after calcitriol treatment are consistent with other research (Cui et al., 2015). However, these effects of calcitriol may be due to direct regulation by local VDR activation, nondirect signaling pathways, or a combination of both. Research by others (Jiang et al., 2014; Kaneko et al., 2015; Patrick and Ames, 2015) has shown roles for vitamin D3 signaling in regulating genes involved in other neurotransmitters, such as glutamate decarboxylase 67 and tryptophan hydroxylase 2; therefore, it is possible that these neurotransmitters could alter dopamine-related gene expression and signaling. Additional studies are necessary to identify the direct gene targets of calcitriol signaling most relevant to behavioral responses.

In addition to expression on dopamine neurons of the midbrain, we observed VDR in the neurons of the dorsal and ventral striatum, suggesting multiple ways to influence dopamine signaling. In fact, since VDR is broadly expressed throughout the brain (Stumpf and O'Brien, 1987; Eyles et al., 2005), it is possible that the behavioral effects are mediated by multiple target regions, including feeding centers such as the hypothalamus. However, the differential effects of calcitriol on *Drd2* expression between the accumbens and dorsal/lateral striatum regions suggest regional differences of VDR target gene regulation. The presence of VDR on these neurons, the acute effects of calcitriol on gene regulation and dopamine release, as well as the rapid effects on locomotor activity are consistent with local VDR activation in the midbrain neurons. Additionally, we observed a significant increase in amphetamine-induced locomotor activity as rapidly as 2 h after calcitriol treatment (data not shown), which supports the contribution of direct signaling to these results. Regardless, we cannot presently exclude possible indirect effects of vitamin D3 on dopamine circuits. Further research is required to investigate the underlying mechanisms in both scenarios.

Previous studies have determined that the dopamine circuits regulating drug seeking and other behaviors, such as feeding, can be influenced by caloric status indirectly through metabolic hormones (Naleid et al., 2005; Fulton et al., 2006; Hommel et al., 2006; Bruijnzeel et al., 2011; Mebel et al., 2012). However, specific roles for dietary micronutrient fluctuations in influencing the function of these circuits in adult animals are not well described. The research presented here expands the potential factors influencing dopamine circuits to include the micronutrient vitamin D3. By reducing the amount of dietary vitamin D3 to resemble levels in common dietary choices observed in obese people (Harnack et al., 2011), we have induced behavioral changes in mice that lead to increased intake and body weight while eating a high fat diet, as well as to increased intake of amphetamine. These increases in consumption are similar to previous clinical observations that obese individuals and drug addicts have similar changes in dopamine circuits (Volkow et al., 2013). It is possible that a chronic dietary D3 deficiency may be another environmental factor that contributes to similar phenotypes via common and relevant neural substrates. The proposed mechanism of action via dopamine circuits also indicates a potential therapeutic use of fully active analogs of vitamin D3 in obesity and drug addiction.

## Synthesis

The decision was a result of the Reviewing Editor Lorna Role and the peer reviewers coming together and discussing their recommendations until a consensus was reached. A fact-based synthesis statement explaining their decision and outlining what is needed to prepare a revision is listed below. The following reviewers agreed to reveal their identity: Emmanuel N. Pothos, David Talmage

Synthesis of reviews for "Vitamin D3: a role in dopamine circuit regulation, diet-induced obesity, and drug consumption" (eN-NWR-0122-15 R1):

Please note that the reviewers' comments have been integrated to reflect their collective impressions, queries, and suggestions for additional revisions:

Summary:

The reviewers agree that the manuscript has been substantively improved by the revisions. In particular, the additional experimental work with standard chow groups was important in addressing prior significant concerns.

Additional minor revisions to be addressed for review by the editor include:

1. It is remarkable that the calcitriol treatment used by the authors does not change calcium levels, at least in serum (figure 2C). Calcitriol is working by increasing the uptake of calcium from the gut into the blood. Beyond any enzymatic deficiencies in obese mice, authors should further clarify any differences in the effects on calcium levels from a standard dose regimen versus what the mice are effectively administered under their procedure.

2. There is still concern about the acute gene expression changes measured in response to calcitriol. Based on the immunostaining in Figure 3, it appears that all, or nearly all cells are positive for VDR whereas only some show presumed VDR responses to calcitriol (e.g. increased Drd2 in nAc and VTA/SN but not lateral or medial striatum). As the authors acknowledge, this could indicate an indirect or complex response to hormone. Alternatively, there might be issues with the antibody used to detect VDR. This latter concern is underscored by the prominent staining of fibers in the VTA / SN. Some mention of specificity controls should be provided.

3. How do the authors account for the fact that food widely available to consumers in Western societies is often fortified in vitamin D, yet the claim is that vitamin D deficiency in Western diets is partly responsible for obesity?

4. There are still several typos and syntax errors throughout the manuscript, please proof read carefully for these

e.g.

"....sacrificed 24 hours lates" line 902

"Other research using obesity-prone rats have demonstrated.....", line 561

"Naïve adult male C57BL/6J group-housed on Std. Chow.....", line 153

## References

[B1] Aguirre Castaneda R, Nader N, Weaver A, Singh R, Kumar S (2012) Response to vitamin D3 supplementation in obese and non-obese Caucasian adolescents. Horm Res Paediatr 78:226-231. 23128469 10.1159/000343446 PMC3557792

[B2] Alkharfy KM, Al-Daghri NM, Yakout SM, Hussain T, Mohammed AK, Krishnaswamy S (2013) Influence of vitamin D treatment on transcriptional regulation of insulin-sensitive genes. Metab Syndr Relat Disord 11:283-288. 10.1089/met.2012.0068 23621113

[B3] Anderson PH, Sawyer RK, May BK, O'Loughlin PD, Morris HA (2007) 25-Hydroxyvitamin D requirement for maintaining skeletal health utilising a Sprague-Dawley rat model. J Steroid Biochem Mol Biol 103:592-595. 10.1016/j.jsbmb.2006.12.086 17267207

[B4] Barchetta I, Carotti S, Labbadia G, Gentilucci UV, Muda AO, Angelico F, Silecchia G, Leonetti F, Fraioli A, Picardi A, Morini S, Cavallo MG (2012) Liver vitamin D receptor, CYP2R1, and CYP27A1 expression: relationship with liver histology and vitamin D3 levels in patients with nonalcoholic steatohepatitis or hepatitis C virus. Hepatology 56:2180-2187. 10.1002/hep.25930 22753133

[B5] Belenchia AM, Tosh AK, Hillman LS, Peterson CA (2013) Correcting vitamin D insufficiency improves insulin sensitivity in obese adolescents: a randomized controlled trial. Am J Clin Nutr 97:774-781. 10.3945/ajcn.112.050013 23407306

[B6] Blum K, Gardner E, Oscar-Berman M, Gold M (2012) “Liking” and “wanting” linked to Reward Deficiency Syndrome (RDS): hypothesizing differential responsivity in brain reward circuitry. Curr Pharm Des 18:113-118. 22236117 10.2174/138161212798919110 PMC3651846

[B7] Brommage R, DeLuca HF (1984) Self-selection of a high calcium diet by vitamin D-deficient lactating rats increases food consumption and milk production. J Nutr 114:1377-1385. 6747721 10.1093/jn/114.8.1377

[B8] Bruijnzeel AW, Corrie LW, Rogers JA, Yamada H (2011) Effects of insulin and leptin in the ventral tegmental area and arcuate hypothalamic nucleus on food intake and brain reward function in female rats. Behav Brain Res 219:254-264. 10.1016/j.bbr.2011.01.020 21255613 PMC3062744

[B9] Calvo MS, Whiting SJ, Barton CN (2004) Vitamin D fortification in the United States and Canada: current status and data needs. Am J Clin Nutr 80:1710S–1716S. 15585792 10.1093/ajcn/80.6.1710S

[B10] Carlsson A (1952) Tracer experiments on the effect of vitamin D on the skeletal metabolism of calcium and phosphorus. Acta Physiol Scand 26:212-220. 10.1111/j.1748-1716.1952.tb00904.x 12985409

[B11] Cashman KD, Kiely M (2016) Tackling inadequate vitamin D intakes within the population: fortification of dairy products with vitamin D may not be enough. Endocrine 51:38-46. 10.1007/s12020-015-0711-x 26260695

[B12] Cass WA, Smith MP, Peters LE (2006) Calcitriol protects against the dopamine- and serotonin-depleting effects of neurotoxic doses of methamphetamine. Ann N Y Acad Sci 1074:261-271. 10.1196/annals.1369.023 17105922

[B13] Cass WA, Peters LE, Fletcher AM, Yurek DM (2012) Evoked dopamine overflow is augmented in the striatum of calcitriol treated rats. Neurochem Int 60:186-191. 10.1016/j.neuint.2011.11.010 22133428 PMC3268902

[B14] Cass WA, Peters LE, Fletcher AM, Yurek DM (2014) Calcitriol promotes augmented dopamine release in the lesioned striatum of 6-hydroxydopamine treated rats. Neurochem Res 39:1467-1476. 10.1007/s11064-014-1331-1 24858239 PMC4125437

[B15] Cekic M, Cutler SM, VanLandingham JW, Stein DG (2011) Vitamin D deficiency reduces the benefits of progesterone treatment after brain injury in aged rats. Neurobiol Aging 32:864-874. 10.1016/j.neurobiolaging.2009.04.017 19482377 PMC3586224

[B16] Chow EC, Quach HP, Vieth R, Pang KS (2013) Temporal changes in tissue 1α,25-dihydroxyvitamin D3, vitamin D receptor target genes, and calcium and PTH levels after 1,25(OH)2D3 treatment in mice. Am J Physiol Endocrinol Metab 304:E977–E989. 10.1152/ajpendo.00489.2012 23482451

[B17] Cui X, Pelekanos M, Liu PY, Burne TH, McGrath JJ, Eyles DW (2013) The vitamin D receptor in dopamine neurons; its presence in human substantia nigra and its ontogenesis in rat midbrain. Neuroscience 236:77-87. 10.1016/j.neuroscience.2013.01.035 23352937

[B18] Cui X, Pertile R, Liu P, Eyles DW (2015) Vitamin D regulates tyrosine hydroxylase expression: N-cadherin a possible mediator. Neuroscience 304:90-100. 10.1016/j.neuroscience.2015.07.048 26210580

[B19] DeLuca HF (1974) Vitamin D: the vitamin and the hormone. Fed Proc 33:2211-2219. 4372106

[B20] Deluca HF, Prahl JM, Plum LA (2011) 1,25-Dihydroxyvitamin D is not responsible for toxicity caused by vitamin D or 25-hydroxyvitamin D. Arch Biochem Biophys 505:226-230. 10.1016/j.abb.2010.10.012 20965147

[B21] Dileone RJ, Taylor JR, Picciotto MR (2012) The drive to eat: comparisons and distinctions between mechanisms of food reward and drug addiction. Nat Neurosci 15:1330-1335. 10.1038/nn.3202 23007187 PMC3570269

[B22] Dodds GS, Cameron HC (1943) Studies on experimental rickets in rats: IV. The relation of rickets to growth, with special reference to the bones. Am J Pathol 19:169-185. 19970680 PMC2033071

[B23] Drincic AT, Armas LA, Van Diest EE, Heaney RP (2012) Volumetric dilution, rather than sequestration best explains the low vitamin D status of obesity. Obesity (Silver Spring) 20:1444-1448. 10.1038/oby.2011.404 22262154

[B24] Eastwood EC, Barkley-Levenson AM, Phillips TJ (2014) Methamphetamine drinking microstructure in mice bred to drink high or low amounts of methamphetamine. Behav Brain Res 272:111-120. 10.1016/j.bbr.2014.06.035 24978098 PMC4167388

[B25] Ersner JS (1940) The treatment of obesity due to dietary indiscretion (overeating) with benzedrine sulfate. Endocrinology 27:776-780. 10.1210/endo-27-5-776

[B26] Eyles DW, Smith S, Kinobe R, Hewison M, McGrath JJ (2005) Distribution of the vitamin D receptor and 1 alpha-hydroxylase in human brain. J Chem Neuroanat 29:21-30. 10.1016/j.jchemneu.2004.08.006 15589699

[B27] Ferreira JG, Tellez LA, Ren X, Yeckel CW, de Araujo IE (2012) Regulation of fat intake in the absence of flavour signalling. J Physiol 590:953-972. 10.1113/jphysiol.2011.218289 22219333 PMC3381321

[B28] Foss YJ (2009) Vitamin D deficiency is the cause of common obesity. Med Hypotheses 72:314-321. 10.1016/j.mehy.2008.10.005 19054627

[B29] Fulton S, Pissios P, Manchon RP, Stiles L, Frank L, Pothos EN, Maratos-Flier E, Flier JS (2006) Leptin regulation of the mesoaccumbens dopamine pathway. Neuron 51:811-822. 10.1016/j.neuron.2006.09.006 16982425

[B30] García OP, Long KZ, Rosado JL (2009) Impact of micronutrient deficiencies on obesity. Nutr Rev 67:559-572. 10.1111/j.1753-4887.2009.00228.x 19785688

[B31] Geiger BM, Behr GG, Frank LE, Caldera-Siu AD, Beinfeld MC, Kokkotou EG, Pothos EN (2008) Evidence for defective mesolimbic dopamine exocytosis in obesity-prone rats. FASEB J 22:2740-2746. 10.1096/fj.08-110759 18477764 PMC2728544

[B32] Geiger BM, Haburcak M, Avena NM, Moyer MC, Hoebel BG, Pothos EN (2009) Deficits of mesolimbic dopamine neurotransmission in rat dietary obesity. Neuroscience 159:1193-1199. 10.1016/j.neuroscience.2009.02.007 19409204 PMC2677693

[B33] Ginde AA, Liu MC, Camargo CA Jr (2009) Demographic differences and trends of vitamin D insufficiency in the US population, 1988-2004. Arch Intern Med 169:626-632. 10.1001/archinternmed.2008.604 19307527 PMC3447083

[B34] Groves NJ, Kesby JP, Eyles DW, McGrath JJ, Mackay-Sim A, Burne TH (2013) Adult vitamin D deficiency leads to behavioural and brain neurochemical alterations in C57BL/6J and BALB/c mice. Behav Brain Res 241:120-131. 10.1016/j.bbr.2012.12.001 23238039

[B35] Gu JM, Xiao WJ, He JW, Zhang H, Hu WW, Hu YQ, Li M, Liu YJ, Fu WZ, Yu JB, Gao G, Yue H, Ke YH, Zhang ZL (2009) Association between VDR and ESR1 gene polymorphisms with bone and obesity phenotypes in Chinese male nuclear families. Acta Pharmacol Sin 30:1634-1642. 10.1038/aps.2009.169 19960008 PMC4007503

[B36] Harnack LJ, Steffen L, Zhou X, Luepker RV (2011) Trends in vitamin D intake from food sources among adults in the Minneapolis-St Paul, MN, metropolitan area, 1980-1982 through 2007-2009. J Am Diet Assoc 111:1329-1334. 10.1016/j.jada.2011.06.009 21872696 PMC3183996

[B37] Hommel JD, Trinko R, Sears RM, Georgescu D, Liu ZW, Gao XB, Thurmon JJ, Marinelli M, DiLeone RJ (2006) Leptin receptor signaling in midbrain dopamine neurons regulates feeding. Neuron 51:801-810. 10.1016/j.neuron.2006.08.023 16982424

[B38] Jeffery RW, Baxter J, McGuire M, Linde J (2006) Are fast food restaurants an environmental risk factor for obesity? Int J Behav Nutr Phys Act 3:2. 10.1186/1479-5868-3-2 16436207 PMC1397859

[B39] Jiang P, Zhang LH, Cai HL, Li HD, Liu YP, Tang MM, Dang RL, Zhu WY, Xue Y, He X (2014) Neurochemical effects of chronic administration of calcitriol in rats. Nutrients 6:6048-6059. 10.3390/nu6126048 25533012 PMC4277014

[B40] Johnson PM, Kenny PJ (2010) Dopamine D2 receptors in addiction-like reward dysfunction and compulsive eating in obese rats. Nat Neurosci 13:635-641. 10.1038/nn.2519 20348917 PMC2947358

[B41] Kamens HM, Burkhart-Kasch S, McKinnon CS, Li N, Reed C, Phillips TJ (2005) Sensitivity to psychostimulants in mice bred for high and low stimulation to methamphetamine. Genes Brain Behav 4:110-125. 10.1111/j.1601-183X.2004.00101.x 15720407

[B42] Kaneko I, Sabir MS, Dussik CM, Whitfield GK, Karrys A, Hsieh JC, Haussler MR, Meyer MB, Pike JW, Jurutka PW (2015) 1,25-Dihydroxyvitamin D regulates expression of the tryptophan hydroxylase 2 and leptin genes: implication for behavioral influences of vitamin D. FASEB J 29:4023-4035. 10.1096/fj.14-269811 26071405

[B43] Kesby JP, Cui X, O'Loan J, McGrath JJ, Burne TH, Eyles DW (2010) Developmental vitamin D deficiency alters dopamine-mediated behaviors and dopamine transporter function in adult female rats. Psychopharmacology 208:159-168. 10.1007/s00213-009-1717-y 19921153

[B44] Keskitalo K, Tuorila H, Spector TD, Cherkas LF, Knaapila A, Kaprio J, Silventoinen K, Perola M (2008) The Three-Factor Eating Questionnaire, body mass index, and responses to sweet and salty fatty foods: a twin study of genetic and environmental associations. Am J Clin Nutr 88:263-271. 18689360 10.1093/ajcn/88.2.263

[B45] Koob GF, Le Moal M (1997) Drug abuse: hedonic homeostatic dysregulation. Science 278:52-58. 9311926 10.1126/science.278.5335.52

[B46] Lagishetty V, Misharin AV, Liu NQ, Lisse TS, Chun RF, Ouyang Y, McLachlan SM, Adams JS, Hewison M (2010) Vitamin D deficiency in mice impairs colonic antibacterial activity and predisposes to colitis. Endocrinology 151:2423-2432. 10.1210/en.2010-0089 20392825 PMC2875827

[B47] Liebman M, Pelican S, Moore SA, Holmes B, Wardlaw MK, Melcher LM, Liddil AC, Paul LC, Dunnagan T, Haynes GW (2003) Dietary intake, eating behavior, and physical activity-related determinants of high body mass index in rural communities in Wyoming, Montana, and Idaho. Int J Obes Relat Metab Disord 27:684-692. 10.1038/sj.ijo.0802277 12833112

[B48] Livak KJ, Schmittgen TD (2001) Analysis of relative gene expression data using real-time quantitative PCR and the 2(-delta delta C(T)) method. Methods 25:402-408. 10.1006/meth.2001.1262 11846609

[B49] Mallya SM, Corrado KR, Saria EA, Yuan FF, Tran HQ, Saucier K, Atti E, Tetradis S, Arnold A (2016) Modeling vitamin D insufficiency and moderate deficiency in adult mice via dietary cholecalciferol restriction. Endocr Res 1-10. 26906176 10.3109/07435800.2016.1141937 PMC4995161

[B50] Mason C, Xiao L, Imayama I, Duggan C, Wang CY, Korde L, McTiernan A (2014) Vitamin D3 supplementation during weight loss: a double-blind randomized controlled trial. Am J Clin Nutr 99:1015-1025. 10.3945/ajcn.113.073734 24622804 PMC3985208

[B51] McDonnell DP, Scott RA, Kerner SA, O'Malley BW, Pike JW (1989) Functional domains of the human vitamin D3 receptor regulate osteocalcin gene expression. Mol Endocrinol 3:635-644. 10.1210/mend-3-4-635 2542779

[B52] McGill AT, Stewart JM, Lithander FE, Strik CM, Poppitt SD (2008) Relationships of low serum vitamin D3 with anthropometry and markers of the metabolic syndrome and diabetes in overweight and obesity. Nutr J 7:4. 10.1186/1475-2891-7-4 18226257 PMC2265738

[B53] Mebel DM, Wong JC, Dong YJ, Borgland SL (2012) Insulin in the ventral tegmental area reduces hedonic feeding and suppresses dopamine concentration via increased reuptake. Eur J Neurosci 36:2336-2346. 10.1111/j.1460-9568.2012.08168.x 22712725 PMC5239666

[B54] Mokdad AH, Ford ES, Bowman BA, Dietz WH, Vinicor F, Bales VS, Marks JS (2003) Prevalence of obesity, diabetes, and obesity-related health risk factors, 2001. JAMA 289:76-79. 12503980 10.1001/jama.289.1.76

[B55] Naleid AM, Grace MK, Cummings DE, Levine AS (2005) Ghrelin induces feeding in the mesolimbic reward pathway between the ventral tegmental area and the nucleus accumbens. Peptides 26:2274-2279. 10.1016/j.peptides.2005.04.025 16137788

[B56] Nicolaysen R (1937) Studies upon the mode of action of vitamin D: the influence of vitamin D on the absorption of calcium and phosphorus in the rat. Biochem J 31:122-129. 16746301 10.1042/bj0310122 PMC1266900

[B57] Pakkala S, de Vos S, Elstner E, Rude RK, Uskokovic M, Binderup L, Koeffler HP (1995) Vitamin D3 analogs: effect on leukemic clonal growth and differentiation, and on serum calcium levels. Leuk Res 19:65-72. 7837819 10.1016/0145-2126(94)00065-i

[B58] Patrick RP, Ames BN (2015) Vitamin D and the omega-3 fatty acids control serotonin synthesis and action, part 2: relevance for ADHD, bipolar disorder, schizophrenia, and impulsive behavior. FASEB J 29:2207-2222. 10.1096/fj.14-268342 25713056

[B59] Paxinos G, Franklin KBJ (2004) The mouse brain in stereotaxic coordinates, Ed 2 San Diego, CA: Academic.

[B79] Paxinos G, Watson C (2007) The rat brain in stereotaxic coordinates, 6th Edition Amsterdam, Boston, Academic Press/Elsevier.

[B60] Pereira MA, Kartashov AI, Ebbeling CB, Van Horn L, Slattery ML, Jacobs DR Jr, Ludwig DS (2005) Fast-food habits, weight gain, and insulin resistance (the CARDIA study): 15-year prospective analysis. Lancet 365:36-42. 10.1016/S0140-6736(04)17663-0 [Mismatch] 15639678

[B61] Ren X, Ferreira JG, Zhou L, Shammah-Lagnado SJ, Yeckel CW, de Araujo IE (2010) Nutrient selection in the absence of taste receptor signaling. J Neurosci 30:8012-8023. 10.1523/JNEUROSCI.5749-09.2010 20534849 PMC6632684

[B62] Robinson TE, Berridge KC (2008) Review. The incentive sensitization theory of addiction: some current issues. Philos Trans R Soc Lond B Biol Sci 363:3137-3146. 10.1098/rstb.2008.0093 18640920 PMC2607325

[B63] Smith JE, Goodman DS (1971) The turnover and transport of vitamin D and of a polar metabolite with the properties of 25-hydroxycholecalciferol in human plasma. J Clin Invest 50:2159-2167. 10.1172/JCI106710 4330006 PMC292150

[B64] Speakman J, Hambly C, Mitchell S, Król E (2008) The contribution of animal models to the study of obesity. Lab Anim 42:413-432. 10.1258/la.2007.006067 18782824

[B65] Stumpf WE, O'Brien LP (1987) 1,25 (OH)2 vitamin D3 sites of action in the brain. An autoradiographic study. Histochemistry 87:393-406. 2828283 10.1007/BF00496810

[B66] Sulzer D, Maidment NT, Rayport S (1993) Amphetamine and other weak bases act to promote reverse transport of dopamine in ventral midbrain neurons. J Neurochem 60:527-535. 8419534 10.1111/j.1471-4159.1993.tb03181.x

[B67] Trinko R, Sears RM, Guarnieri DJ, DiLeone RJ (2007) Neural mechanisms underlying obesity and drug addiction. Physiol Behav 91:499-505. 10.1016/j.physbeh.2007.01.001 17292426

[B68] Tsukada H, Harada N, Nishiyama S, Ohba H, Kakiuchi T (2000) Dose-response and duration effects of acute administrations of cocaine and GBR12909 on dopamine synthesis and transporter in the conscious monkey brain: PET studies combined with microdialysis. Brain Res 860:141-148. 10727633 10.1016/s0006-8993(00)02057-6

[B69] U.S. Department of Agriculture (2015) USDA National Nutrient Database for Standard Reference Release 28, pp 1-149. Beltsville, MD: United States Department of Agriculture Nutrient Data Laboratory.

[B70] Van Heek M, Compton DS, France CF, Tedesco RP, Fawzi AB, Graziano MP, Sybertz EJ, Strader CD, Davis HR Jr (1997) Diet-induced obese mice develop peripheral, but not central, resistance to leptin. J Clin Invest 99:385-390. 10.1172/JCI119171 9022070 PMC507810

[B71] Vanderschuren LJ, Pierce RC (2010) Sensitization processes in drug addiction. Curr Top Behav Neurosci 3:179-195. 10.1007/7854_2009_21 21161753

[B72] Vasilopoulos Y, Sarafidou T, Kotsa K, Papadimitriou M, Goutzelas Y, Stamatis C, Bagiatis V, Tsekmekidou X, Yovos JG, Mamuris Z (2013) VDR TaqI is associated with obesity in the Greek population. Gene 512:237-239. 10.1016/j.gene.2012.10.044 23103831

[B73] Volkow ND, Wang GJ, Tomasi D, Baler RD (2013) Obesity and addiction: neurobiological overlaps. Obes Rev 14:2-18. 10.1111/j.1467-789X.2012.01031.x 23016694 PMC4827343

[B74] Vucetic Z, Reyes TM (2010) Central dopaminergic circuitry controlling food intake and reward: implications for the regulation of obesity. Wiley Interdiscip Rev Syst Biol Med 2:577-593. 10.1002/wsbm.77 20836049

[B75] Wang GJ, Volkow ND, Logan J, Pappas NR, Wong CT, Zhu W, Netusil N, Fowler JS (2001) Brain dopamine and obesity. Lancet 357:354-357. 11210998 10.1016/s0140-6736(00)03643-6

[B76] Wortsman J, Matsuoka LY, Chen TC, Lu Z, Holick MF (2000) Decreased bioavailability of vitamin D in obesity. Am J Clin Nutr 72:690-693. 10966885 10.1093/ajcn/72.3.690

[B77] Yin Y, Yu Z, Xia M, Luo X, Lu X, Ling W (2012) Vitamin D attenuates high fat diet-induced hepatic steatosis in rats by modulating lipid metabolism. Eur J Clin Invest 42:1189-1196. 10.1111/j.1365-2362.2012.02706.x 22958216

[B78] Young KA, Engelman CD, Langefeld CD, Hairston KG, Haffner SM, Bryer-Ash M, Norris JM (2009) Association of plasma vitamin D levels with adiposity in Hispanic and African Americans. J Clin Endocrinol Metab 94:3306-3313. 10.1210/jc.2009-0079 19549738 PMC2741711

